# Transcriptomic changes and gene fusions during the progression from Barrett’s esophagus to esophageal adenocarcinoma

**DOI:** 10.1186/s40364-024-00623-8

**Published:** 2024-08-07

**Authors:** Yusi Fu, Swati Agrawal, Daniel R. Snyder, Shiwei Yin, Na Zhong, James A. Grunkemeyer, Nicholas Dietz, Ryan Corlett, Laura A. Hansen, Al-Refaie Waddah, Kalyana C. Nandipati, Jun Xia

**Affiliations:** 1 Department of Biomedical Sciences, Creighton University School of Medicine, Omaha, NE USA; 2 Department of Surgery, Creighton University School of Medicine, Omaha, NE USA; 3 Department of Pathology, Creighton University School of Medicine, Omaha, NE USA

**Keywords:** Barrett’s esophagus, Esophageal adenocarcinoma, Keratin, Cell identity, RNA-seq, Gene fusion, Cancer

## Abstract

The incidence of esophageal adenocarcinoma (EAC) has surged by 600% in recent decades, with a dismal 5-year survival rate of just 15%. Barrett’s esophagus (BE), affecting about 2% of the population, raises the risk of EAC by 40-fold. Despite this, the transcriptomic changes during the BE to EAC progression remain unclear. Our study addresses this gap through comprehensive transcriptomic profiling to identify key mRNA signatures and genomic alterations, such as gene fusions. We performed RNA-sequencing on BE and EAC tissues from 8 individuals, followed by differential gene expression, pathway and network analysis, and gene fusion prediction. We identified mRNA changes during the BE-to-EAC transition and validated our results with single-cell RNA-seq datasets. We observed upregulation of keratin family members in EAC and confirmed increased levels of keratin 14 (KRT14) using immunofluorescence. More differentiated BE marker genes are downregulated during progression to EAC, suggesting undifferentiated BE subpopulations contribute to EAC. We also identified several gene fusions absent in paired BE and normal esophagus but present in EAC. Our findings are critical for the BE-to-EAC transition and have the potential to promote early diagnosis, prevention, and improved treatment strategies for EAC.

**Supplementary Information:**

The online version contains supplementary material available at 10.1186/s40364-024-00623-8.

## To the editor

**Fig. 1:**
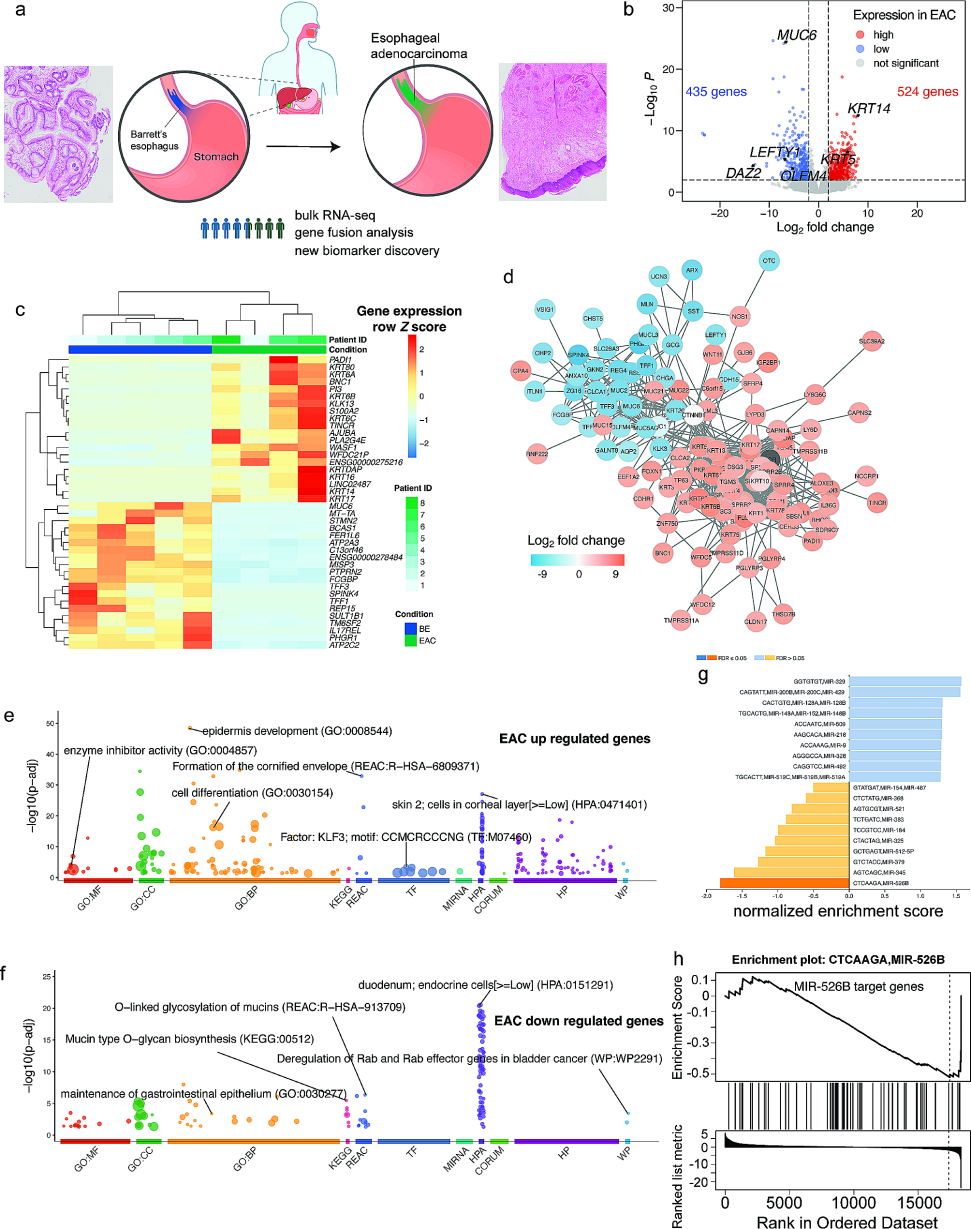
The transcriptome and functional changes during the transition from Barrett’s esophagus (BE) to esophageal adenocarcinoma (EAC). **a** Schematic representation of EAC development, illustrating the progression from healthy esophageal tissue, characterized by squamous mucosa, to Barrett’s metaplasia—a condition wherein the normal squamous epithelial cells are replaced by columnar cells. This pathological change typically occurs at the junction of the esophagus and stomach and is a known precursor to EAC, depicted as the final stage of this progression. The transformation is indicated by the arrow pointing from the ‘Barrett’s metaplasia’ region to the area labeled as ‘esophageal adenocarcinoma,’ demonstrating the location and potential spread of the disease. Example slides with hematoxylin and eosin (HE) staining are also provided to visualize these changes. **b** Volcano plot of differentially expressed genes (DEGs) detected when comparing EAC to BE; scatter plot shows log2 fold-change (EAC/BE) vs. ‒log10 adjusted *p*-value for gene expression data. Points represent individual genes, with statistically significant DEGs highlighted (red, upregulated in EAC; blue, downregulated in EAC). Key genes of interest are labeled (e.g., *MUC5B*, *KRT14*). **c** Heatmap indicating expression levels of top DEGs ranked on adjusted *p*-value across different conditions and samples. Color scale (blue to red) indicates expression level from low to high normalized to z-score for each gene, respectively. Clusters of genes and conditions are annotated on the axes. BE, cyan; EAC, purple. **d** Network diagram showing the protein–protein interaction network of DEGs with |log2(FC)| > 5 in BE vs. EAC; red nodes represent upregulated genes, and blue nodes represent downregulated genes. The degrees of interaction for all DEGs were calculated with Cytoscape, genes with higher fold changes are shown in darker colors. Nodes represent genes, and lines (edges) indicate interactions. Genes shown in grey are not DEGs but are connected to the network genes. (**e**,**f**) Visualization of results from functional enrichment analysis with differentially expressed genes (DEGs) upregulated (e) and downregulated (f) in EAC relative to BE. Enriched terms are categorized by gene ontology (GO), including molecular function (MF), cellular component (CC), and biological process (BP), Additionally, regulatory motifs, and results from other protein databases are included. The *y*-axis represents the ‒log10 *p*-value, indicating the statistical significance of each term. Key developmental processes, such as epidermis development and cell differentiation, are prominently featured among upregulated genes, along with KLF3 transcription factor motif enrichment. Downregulated genes indicate a decrease in processes such as Mucin type O–glycan biosynthesis and the maintenance of the gastrointestinal epithelium, indicating a trend toward dedifferentiation in EAC. **g** Graphical representation of gene set enrichment analysis (GSEA) results, showing microRNAs (miRNAs) with significant enrichment scores. The normalized enrichment scores for each miRNA are displayed, highlighting those with potential roles in the BE-to-EAC transition. **h** Detailed GSEA results for miR-526B; enrichment plot shows the distribution of miR-526B target genes across a ranked list of all genes, with a focus on those that are downregulated during BE to EAC progression. The bar graph below indicates the rank of the target genes, reflecting their relevance to the disease process

Esophageal adenocarcinoma (EAC) is associated with a low overall 5-year survival of 15%. The incidence of EAC increased by 600% over the past four decades, yet the underlying causes are still not fully understood [1]. Barrett’s esophagus (BE) [2], is identified as a precursor to EAC [3], elevating EAC risk by 40-fold [4]. However, transcriptomic alterations and gene fusions during the progression from BE to EAC remain limited [5, 6]. Here, we performed a comprehensive RNA-seq analysis with immunofluorescence validation, followed by gene fusion prediction and long-reads validation, from patients with EAC, BE, or concurrent BE/EAC to delineate the molecular changes occurring during the BE-to-EAC transition (Fig. 1a, S1, Material and Methods). Our findings reveal promising biomarkers that could inform targeted therapies and diagnostic tools for early detection, thereby improving patient outcomes.

When comparing transcriptomic profiles in EAC samples with those in BE samples, we identified 524 significantly upregulated and 435 significantly downregulated genes (Fig. 1b and Table S1). Heatmap illustrating expression levels of the top 40 genes showing consistent changes across sample groups is shown in Fig. 1c. Network analysis was then performed to identify interactions between these DEGs (Fig. 1d and Fig. S2). From these analyses, we identified keratin family members as hub genes that interact with the highest number of DEGs, suggesting a major role for keratins in the progression from BE to EAC (Fig. S3).

To assess functional changes occurring during the transition from BE to EAC, we performed Gene Ontology (GO) functional enrichment analysis with significantly DEGs. We further analyzed biological pathways using the Kyoto Encyclopedia of Genes and Genomes (KEGG), Reactome, and WikiPathways. In addition, to gain a more comprehensive functional enrichment for the genes, we identified regulatory motifs for transcription factor (TRANSFAC) and microRNA (miRTarBase) binding, analyzed the Human Protein Atlas and CORUM databases, and assessed human phenotype ontology (Table S2). For upregulated genes, we identified development and differentiation-related changes and enrichment of transcription factor KLF3 targets (Fig. 1e). For the downregulated genes, we identified Mucin type O − glycan biosynthesis and duodenum endocrine cells (Fig. 1f), indicating dedifferentiation during the BE-to-EAC transition.

We next performed GSEA for putative miRNA targets to identify potential regulators of the switch from BE to EAC (Fig. 1g). Notably, miR-526B, exhibited a significant false-discovery rate (FDR) (Fig. 1h). In prior studies, miR-526B was shown to suppress cell proliferation, cell invasion, and the epithelial-to-mesenchymal transition in breast cancer by targeting TWIST1 [7]. Here, we found that targets for miR-526B downregulated during the BE-to-EAC transition include deleted in azoospermia (DAZ)1–4, aristaless-related homeobox (ARX), and hedgehog-interacting protein (HHIP). These targets further indicate dedifferentiation in the switch from BE to EAC, in part, via potential miRNA regulation. Overall, the upregulation of genes involved in development and differentiation in EAC may indicate a dedifferentiation process, which is a common hallmark of many cancers. Conversely, the downregulation of genes associated with mucin biosynthesis and specific cellular functions in the duodenum might reflect a loss of typical epithelial characteristics or functions.

**Fig. 2:**
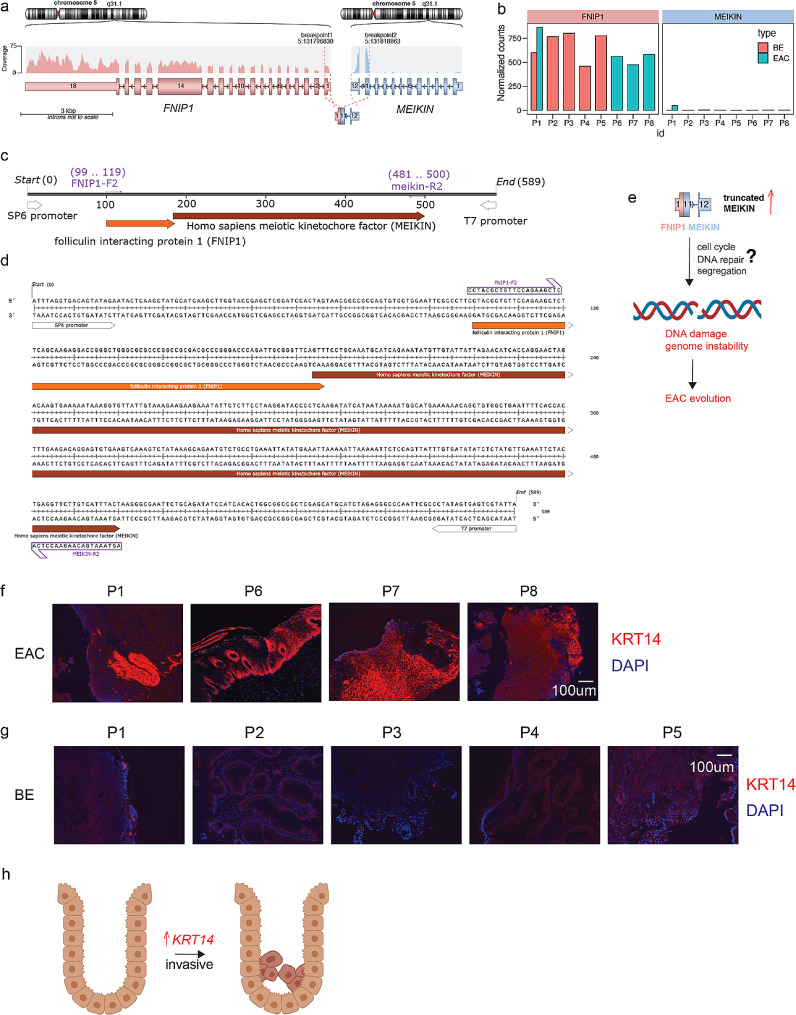
Analysis and validation of the predicted *FNIP1–MEIKIN* fusion, and elevated KRT14 protein levels in esophageal adenocarcinoma (EAC) compared to Barrett’s esophagus (BE). **a** Genomic mapping and expression profiling for the potential *FNIP1–MEIKIN* fusion. The top panel shows the chromosome 5 bands with the positions of the *FNIP1* and *MEIKIN* genes indicated. The middle panel illustrates the expression levels across exons of both genes, with a higher density of reads suggesting increased expression. A detailed exon structure for *FNIP1* and *MEIKIN* is provided, with the direction of transcription indicated by arrows. Dotted lines between the genes indicate the predicted fusion points based on RNA-seq data. **b** Bar chart showing *FNIP1* and *MEIKIN* expression levels in BE and/or EAC tissue obtained from eight patients (P1–P8). Elevated expression of *MEIKIN* is observed in patient P1, potentially resulting from a *FNIP1–MEIKIN* gene fusion. The *y*-axis denotes normalized expression counts, and patient IDs are shown on the *x*-axis. **c** The fusion gene structure as indicated by whole-plasmid sequencing. The *FNIP1* gene (orange) was shown to be directly fused with the *MEIKIN* gene by the FNIP1–F2 and MEIKIN–R2 primer pairs. **d** Detailed sequences of the *FNIP1–MEIKIN* fusion illustrating the junction of the fused genes. **e** Proposed model: *FNIP1–MEIKIN* fusion re-activates the meiosis gene *MEIKIN*, potentially leads to chromosome segregation defects, cell cycle dysregulation, and DNA damage [11]. Excess DNA damage promotes mutagenesis, and drive cancer evolution [12]. **f** Immunofluorescence (IF) staining for keratin 14 (KRT14) in EAC samples from Patients 1, 6, 7, and 8. *KRT14* expression is markedly elevated in EAC samples. **g** IF staining for KRT14 in BE samples from patients shown in P1-5, revealing low expression of *KRT14* and highlighting the difference in protein expression profiles between EAC and BE tissues. **h** Proposed model: *KRT14* upregulation promotes invasion and migration of the cells during EAC development

We further analyzed gene events from our cohort, including one paired BE and EAC case. Most gene fusions detected were patient-specific (Table S3) and did not overlap with an earlier study [8]. In Patient 1, we found that the promoter of the *FNIP1* gene was fused to the adjacent gene *MEIKIN* (Fig. 2a). *FNIP1* was overexpressed in all samples, whereas *MEIKIN* was not expressed in most cases (Fig. 2b). However, in this patient, fusion between the last two exons of *MEIKIN* and the strong *FNIP1* promoter led to elevated *MEIKIN* expression (Fig. 2c and d). Importantly, we validated this fusion event using RT-PCR and nanopore sequencing and confirmed that it was not present in the individual’s paired BE sample (Fig. S4), indicating the event occurred during the progression to EAC. We proposed that this FNIP1-MEIKIN fusion reactivates the meiosis gene(s) and promote genome instability and cancer (Fig. 2e). Other gene fusions predicted, including CCAT1–CASC8 and SPAG1–CA10, were also validated by RT-PCR and sequencing (Fig. S5, S6, Table S3, S4). These fusions were also either exclusively found or present at a higher abundance in EAC compared to BE suggesting that they might drive the BE-to-EAC transition. Although it is unclear which fusion(s) mechanistically drove the transition, the increase of gene fusions in general could be considered as a biomarker for BE-to-EAC transition.

Lastly, we performed IF staining to validate the elevated expression of keratin family members in EAC tissue relative to BE tissue. Although numerous KRT proteins showed increased expression in our EAC RNA-seq dataset, we chose KRT14 as it was one of the most highly abundant proteins. H&E and IF staining revealed characteristic histology and elevated expression of *KRT14* in EAC samples (Fig. 2f, S7a-b). The increased *KRT14* expression was primarily localized in adenocarcinoma to in all cases. In contrast, BE samples showed distinctly lower expression of *KRT14* throughout the tissue (Fig. 2g, S7c-d). We speculated that *KRT14* upregulation promotes cellular behaviors such as invasion and migration during EAC development (Fig. 2h). Similar findings were observed in other cancers such as lung cancer [9], implying a general mechanism of action by *KRT14* upregulation. Lastly, survival analysis found that high-*KRT14* did not predict poor survivals but there are other biomarkers from our study that can predict patient outcomes (Fig. S8).

In summary, we conducted RNA-seq analysis and identified alterations in mRNA expression that occur during the transition from BE to EAC. Interestingly, we observed significant changes of the keratin genes. These genes are crucial, as they play a vital role in the structural integrity and function of epithelial cells, which might be an important problem in the EAC pathogenesis. We further identified several oncogene fusions that may involve in the transition from BE to EAC. Oncogene fusions are mutational events wherein parts of two different genes are merged to create a new hybrid gene, often leading to aberrant cell growth and cancer development [10]. Identification of these fusions present in EAC has the potential to further our understanding of the molecular mechanisms underlying the progression of this disease and may open new avenues of research for the development of targeted therapies (Supplement text 1).

### Electronic supplementary material

Below is the link to the electronic supplementary material.

Supplementary Material 1

Supplementary Material 2

Supplementary Material 3

Supplementary Material 4

Supplementary Material 5

## Data Availability

The raw fastq.gz files can be found at https://www.ncbi.nlm.nih.gov/bioproject/1106179. We have uploaded all the data to SRA under the accession number SRA. PRJNA945944 (EAC) and PRJNA1106179 (BE).

## Declarations

### Code availability

Code for these analyses will be made available to qualified researchers on reasonable request from the corresponding author.

## Abbreviations

BE: Barrett’s esophagus
EAC: Esophageal adenocarcinoma
RNA-seq: RNA sequencing
scRNA-seq: Single-cell RNA sequencing
KRT14: Keratin 14
miRNA: MicroRNA
GO: Gene Ontology
KEGG: Kyoto Encyclopedia of Genes and Genomes
GSEA: Gene set enrichment analysis
H&E: Hematoxylin and eosin
IF: Immunofluorescence
DAPI: 4’,6-diamidino-2-phenylindole
DEG: Differentially expressed gene
FDR: False-discovery rate
DAZ1/2/3/4: Deleted in azoospermia 1/2/3/4
ARX: Aristaless-related homeobox
HHIP: Hedgehog-interacting protein
PCR: Polymerase chain reaction
PCA: Principal component analysis
RT-PCR: Reverse transcription PCR
TCGA: The Cancer Genome Atlas
UCSC: University of California Santa Cruz
PFI: Progression-free Interval
