# Supplementary material for: Transcriptomic changes and gene fusions during the progression from Barrett’s esophagus to esophageal adenocarcinoma
Source: Biomark Res. 2024 Aug 7;12:78. doi: 10.1186/s40364-024-00623-8 (PMC11304724; doi:10.1186/s40364-024-00623-8)
Supplement: Supplementary file 1 — Supplementary Material 1 [file 40364_2024_623_MOESM1_ESM.pdf]

Supplemental Material 1

Material and methods

Study subjects

This study was conducted after receiving approval from the Institutional Review Board (IRB) of Creighton University (IRB No. 1194896-3). Patients were recruited from the surgery clinics at Creighton University Medical Center and CHI Health Immanuel Medical Center, and informed consent was obtained from all participants. Patients ages 19 years or older with a clinical diagnosis of BE or EAC confirmed by endoscopic inspection of the esophagus complemented by esophageal histology were included. Exclusion criteria included those ages 18 years or younger, individuals unwilling to participate in the study, and patients with biopsies that did not confirm EAC. Biopsy samples were collected from BE and EAC lesions and subjected to detailed pathological assessment for confirmation of BE or EAC diagnosis. In cases of concurrent BC and EAC, biopsy was taken from both BE and EAC lesions.

Sample collection and storage

EAC and BE tissues were collected during surgical endoscopy or surgical resection. All freshly endoscopic biopsies and samples from the resected biopsies tissues were divided into two parts; one part was subjected to RNA extraction for RNA-seq, and the other was formalin-fixed and embedded in paraffin for pathological grading and immunofluorescence (IF) staining. All tissue slides were assessed independently by another pathologist, and only those samples matching previous diagnoses were processed for RNA-seq. After collection, tissue samples were placed in RNAlater (Thermo Fisher Scientific) to stabilize and protect cellular RNA and stored at –20°C. All samples were processed within 24 h to ensure preservation of RNA quality. Results from EAC and normal tissue RNA-seq were published previously [1], and the data were deposited in the Gene Expression Omnibus (GEO; PRJNA945944, EAC and normal), BE (PRJNA 1106179).

RNA extraction, RNA-seq library preparation, and next-generation sequencing

As noted above, tissues were preserved in RNAlater solution prior to total RNA extraction, which was performed with TRIzol Reagent (Invitrogen), according to the manufacturer's provided protocol. RNA quality and quantity were assessed by NanoDrop (Thermo Fisher Scientific) and Agilent Bioanalyzer 2100. Samples with an RNA integrity number (RIN) >6 were subjected to downstream library preparation. For each sample, 1µg of total RNA was used as input to construct a stranded RNA-seq library using the Universal Plus mRNA-Seq with NuQuant (Tecan Genomics) protocol. RNA-seq analysis was performed on an Illumina Nextseq550 sequencer with 76-bp paired-end reads. For each sample, we obtained at least 20 million sequencing reads to ensure comprehensive transcriptomic profiling.

Data analysis

Gene counts

We used the nf-core/rnaseq pipeline [2](version 3.12.0) to determine gene counts in each sample. Briefly, all FastQ files from a sample were merged, and read quality was assessed with FastQC; adapter and low-quality reads were then trimmed with Trim Galore. Genome contaminants were removed with BBSplit, and ribosomal RNAs were removed with SortMeRNA. Reads were aligned to the human reference genome GRCh38 using STAR [3], with GENCODE release V44 gene annotations, and Salmon [4] was used to calculate transcript-level abundances and gene counts. General quality control metrics were shown in **Figure S1**.

Differential gene expression analysis

R software (v4.2.1) was used to identify differentially expressed genes (DEGs) from pair-wise comparisons of BE and EAC patients. Transcript-level abundances obtained from Salmon [4] were imported into R with tximport, which estimates counts based on transcript lengths and

summarizes the data into matrices for use in downstream gene-level analysis. Differential expression analysis was performed with DESeq2 [5], which generates results tables containing log2 fold-changes, *p*-values from the Wald test, and adjusted *p*-values for each comparison. For visualization and ranking of DEGs, we used the log2 fold-change after applying shrinkage with empirical Bayes shrinkage estimators for effect sizes. Genes with adjusted $p < 0.01$ and log2(fold-change) > 2 were identified as significantly upregulated genes, and those with adjusted $p < 0.01$ and log2(fold change) < –2 were identified as significantly downregulated genes. The gene identities and statistics from DEG analysis were in **Table S1**.

Network analysis

Protein–protein interaction networks for all DEGs were assessed and visualized with Cytoscape [6] (**Figure 1** and **Figure S2**). The STRING database [7] was first used to query protein–protein interactions and construct the networks. DEGs were then imported into Cytoscape, and Cytoscape Automation py2cytoscape (2019 release) was used to map fold-changes to the color of each node, with red nodes representing upregulated genes and blue nodes representing downregulated genes. The degrees of each gene were calculated with Cytoscape and mapped to the size of the node, with larger nodes representing hub genes that interact with a greater number of genes.

Gene set and Functional enrichment analysis

Gene set enrichment analysis (GSEA) for miRNAs was conducted for all expressed genes ranked by their fold-change when comparing BE to EAC as input for WEB-based Gene SeT AnaLysis Toolkit (WebGestalt) [8], a web-based functional enrichment analysis tool. This analysis was based on motifs in the 3' untranslated region that represent putative target sites for mature human miRNAs from the v7.1 miRBase. Functional enrichment analysis was conducted using g:Profiler, a publicly accessible web server designed to characterize and manipulate gene lists. This analysis

encompasses enriched terms from Gene Ontology (GO) categories such as molecular function (MF), cellular component (CC), and biological process (BP). Additionally, it includes pathways, regulatory motifs, and data from various protein databases (**Table S2**).

Analysis of undifferentiated and differentiated BE markers with DEGs of EAC and BE transcriptomes

To validate our RNA-seq data suggesting the progression to EAC from undifferentiated BE cells and provide further support for our hypothesis that EAC is associated with an additional loss of cell identity, we performed a comparative analysis of DEGs in our dataset against markers associated with both differentiated and undifferentiated BE cells [9]. From this analysis, we identified the overlap between markers and our upregulated and downregulated genes (**Figure S3a**). We observed a greater overlap between differentiated BE markers and our downregulated gene set than was detected for our upregulated genes during the progression to EAC, further indicating that undifferentiated BE subpopulations play a significant role in the development of EAC. Interestingly, we also noted a greater overlap between undifferentiated marker genes and our downregulated gene set (**Figure S3b**; $p < 0.05$), suggesting a loss of cell identity. Of note, upregulated undifferentiated marker genes in our dataset include the gene *OLFM4* (**Figure S3c**), which was identified as a marker gene for undifferentiated cells in BE by a separate scRNA-seq study [10]. In addition, this scRNA-seq analysis revealed a subpopulation of Barrett cells that express high levels of the undifferentiated marker *LEFTY1*, as well as *DAZ1* (y chromosome gene) and *ARX* (MiR-526B target) in **Figure S3c**.

RNA fusion detection

The prevalence of gene fusion events in EAC and potentially BE is another notable aspect of our findings. These events may be key drivers of disease progression, and thus, functional

characterization of EAC-associated gene fusions could provide new insights into disease etiology. However, a previous study reported minimal overlap between gene fusions identified in our study [11], which suggests a broader spectrum of gene fusion events during BE development and the BE-to-EAC transition than previously anticipated. This variability in the literature highlights the complexity of the genomic landscape in EAC and the potential for discovering novel therapeutic targets. Given that gene fusions often result in the production of novel chimeric proteins with functions distinct from their parental genes, the *FNIP1–MEIKIN* fusion (**Figure 2**), if translated, might result in a protein with altered or enhanced functions that could drive the neoplastic process. FNIP1 is known to be involved in energy metabolism, whereas MEIKIN is crucial for meiosis [12], and thus, enhanced expression of the fusion product in EAC may provide a growth or survival advantage to these cells. The nf-core/rna fusion pipeline (version 2.3.4) was used to detect and visualize fusion genes with the following sub-workflows: STAR fusion [13], arriba [14], fusioncatcher [15], kallisto [16], and pizzly [17]. Fusion-report was then used to generate a composite report with all fusions detected by the different tools, and gene-fusion events were visualized in FusionInspector [18]. Fusion events detected by at least two tools and their presence in the Mitelman Database of Chromosome Aberrations and Gene Fusions in Cancer are reported in **Table S3**.

Reverse transcription (RT)-PCR to detect gene fusions

RT-PCR was performed using the SuperScript IV One-Step RT-PCR System (Invitrogen). In brief, template RNA was pretreated with ezDNase (Invitrogen), and 100 ng was added to each 50-μL RT-PCR reaction containing 2× reaction mix, 10-μM forward and reverse primers, and SuperScript IV enzyme. Reactions were run on a BioRad C1000 thermal cycler using the following cycling conditions: 55°C for 10 min; 98°C for 2 min; 35 cycles of 98°C for 10 sec, 60°C for 10 sec, and 72°C for 30 sec per kb; and a final extension at 72°C for 5 min. The primer pairs used in the study include SPAG9-F1 paired with CA10-R1 and SPAG9-F2 paired with CA10-R2 for the

targeting of specific gene sequences. Additionally, to analyze the CASC gene family, CASC19CCAT1-F1 was paired with CASC8-R1, and CCAT1-F2 was combined with CASC8-R2. In another set of combinations, Guk1-F was used in conjunction with USH2A-R1 and USH2A-R2, FNIP1-F1 was paired with ME2K2N-R1, and FNIP1-F2 was matched with MEIKIN-R2, to enable a detailed study of these gene interactions. We also used CCAT1-F1 with CASC8-R1 and CCAT1-F2 with CASC8-R2 for additional analysis of CASC gene sequences. Similarly, GUK1 was consistently paired with USH2A-R1 to assess these gene regions, and FNIP1-F1 and FNIP1-F2 were paired with MEIKINR1 and MEIKIN-R2, respectively, to better understand the interactions between these specific genes. Platinum SuperFi DNA polymerase was used in the amplification. This comprehensive set of primer pairings enabled a diverse and targeted approach to fusion gene analysis (**Figure S4**). The complete list of primers lists used in this study is listed in **Table S4.**

Agarose gel electrophoresis

PCR products were analyzed by electrophoresis on 1% agarose gels containing 1x SYBR™ Green I Nucleic Acid Gel Stain (cat. #S7563). For each lane, 5 µL PCR product was mixed with 1 µL 6× loading dye and run alongside a 1 kb DNA ladder. Gels were run at 135 V for 20-30 min and imaged under UV light.

DNA clean-up and TOPO cloning

PCR products were purified using the DNA Clean & Concentrator kit (Zymo Research) as per the manufacturer's guidelines, and purified products were eluted in 30 µL nuclease-free water. For TOPO Cloning, purified PCR products were cloned into the pCR-Blunt II-TOPO vector using the Zero Blunt TOPO PCR Cloning Kit (Invitrogen). The TOPO cloning reactions contained 4 µL PCR product, 1 µL salt solution, and 1 µL TOPO vector, incubated for 5 min at room temperature before being placed on ice. For transformation into *Escherichia coli*, 2 µL of each TOPO cloning reaction

was added to a vial of NEB® 5-alpha Competent *E. coli* (High Efficiency) and gently mixed. The mixture was incubated on ice for 30 min, subjected to a 30-sec heat shock at 42°C, and then placed back on ice for 2 min. Subsequently, 250 µL of LB media was added, and the cells were incubated with shaking at 37°C for 1 h. Transformed *E. coli* cells were spread on LB agar plates containing 50-µg/mL kanamycin and incubated overnight at 37°C. Screening of individual colonies for the correct inserts was performed by colony PCR, restriction digestion, or whole-plasmid sequencing with the PlasmidSaurus platform, ensuring accurate identification of desired clones.

Whole-plasmid sequencing

Inserted sequences in the TOPO backbone plasmid were identified using the Plasmidsaurus whole-plasmid sequencing method, which was chosen for its ability to provide comprehensive and accurate sequencing of the entire plasmid. Plasmid DNA was prepared using standard protocols and sequenced on the Plasmidsaurus platform. Raw reads were aligned against each other to generate a high-accuracy circular consensus sequence and a set of gene annotations, followed by identification and characterization of the inserted sequences (**Figures S5-6**).

Histological and IF staining

Tissue-staining protocols were described previously [19]. In brief, samples for histological staining (**Figure S7**) were prepared by immersing in formalin for 14 to 18 h, followed by dehydration in 70% alcohol overnight. Subsequently, slides were prepared for hematoxylin and eosin (H&E) automatic staining. Slides were scanned using an Olympus VS120 Slide Scanner, with an initial 4× quick scan, followed by scanning at tissue magnification using a 20× objective lens. For IF analysis (**Figure S7**), formalin-fixed paraffin-embedded (FFPE) samples were subjected to deparaffinization and rehydration using SafeClear and ethanol, as described [20]. Antigen retrieval was then performed using a Tris-based buffer at 98°C in a steamer for 30 min, after

which samples were blocked in a solution of 10% normal goat serum with 3% bovine serum albumin (BSA) in phosphate-buffered saline (PBS), containing 0.1% Triton X-100. Anti-keratin 14 (KRT14) primary antibody (BioLegend, cat. #905304, diluted 1:500 and 1:5000) was prepared in solution containing 3% BSA in PBS with 0.1% Triton X-100 and applied to the slides. Slides were then incubated overnight at room temperature in a humid environment. Secondary antibody (goat anti-rabbit; Invitrogen, cat. #A32740, diluted 1:1000) was also prepared in 3% BSA in PBS with 0.1% Triton X-100 and applied to the slides for a 1-h incubation at room temperature in a humid environment. Nuclei were stained in antifade mounting buffer with 4',6-diamidino-2-phenylindole (DAPI; VectorLabs, cat. #H2000), and imaging was performed using a Nikon Eclipse Ni fluorescence microscope with a 200-ms exposure time for both DAPI and TRITC channels.

TCGA data survival analysis

We downloaded the expression data for the TCGA ESCA study from UCSC Xena [21], along with the associated survival data. To ensure comparability with our dataset, we filtered the TCGA dataset to only include samples with the histological type "Esophagus Adenocarcinoma, NOS," which resulted in a total of 99 individuals.

For the survival analysis, we used the R package 'survival' to analyze the data. The 'survival' package enabled us to compare the survival rates among these individuals, focusing on differences in gene expression levels. In this analysis, "high" expression refers to the top 20% expression of a particular gene in the study populations. Progression-free intervals (PFI) were used as the metric for survival analysis (**Figure S8**).

Supplementary Figures

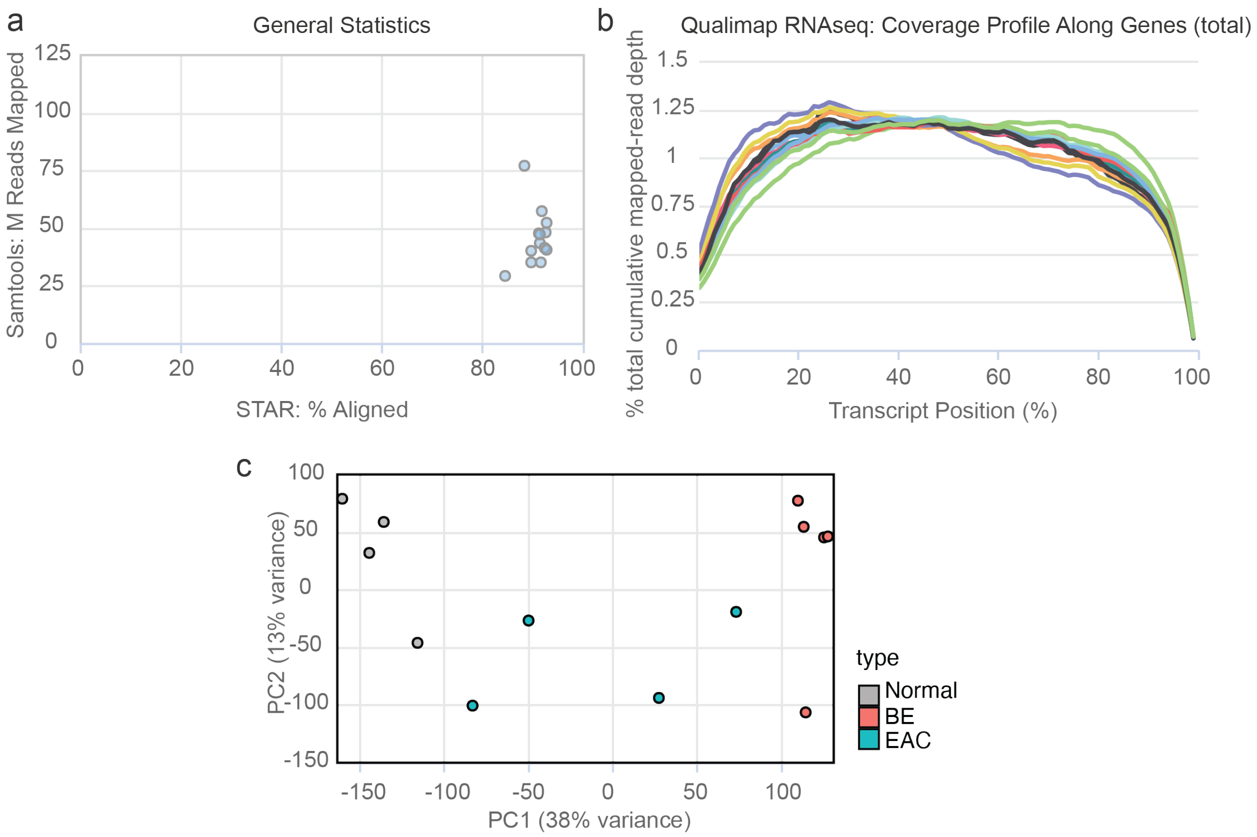

Figure S1: Quality control metrics for RNA sequencing (RNA-seq) datasets and PCA analysis. (a) The scatter plot displays the relationship between the percentage of reads aligned using the STAR aligner (*x*-axis) and the number of reads mapped in millions (*y*-axis) for each RNA-seq sample. Data points above the 80% threshold indicate a high alignment rate, demonstrating the efficiency of the STAR aligner in our dataset. All samples achieved a mapping rate of >80%, with a substantial number of reads (>25 million) mapped to the reference genome, indicating robust data quality and reliability of subsequent analyses. (b) The graph represents the coverage profile across all transcripts in our RNA-seq dataset, with transcript positions displayed as a percentage from the 5' end (0%) to the 3' end (100%) on the *x*-axis. The *y*-axis shows the cumulative mapped read depth as a percentage of the total. Lines of various colors represent individual samples, illustrating a uniform coverage profile across the length of the transcripts. This

uniformity indicates minimal 5’ or 3’ bias, thus ensuring that the RNA-seq data accurately reflect transcript abundance across the entire length of the genes. (c) Principal component plot of the samples based on their RNA-seq results. The figure shows the samples in a 2D plane spanned by their first two principal components, which account for 38% and 13% of the variance, respectively. Samples from the same group are closer to each other, indicating true biological differences based on EAC development stage, rather than random or batch effects. EAC groups tend to have more transcriptional heterogeneity than the BE and normal groups.

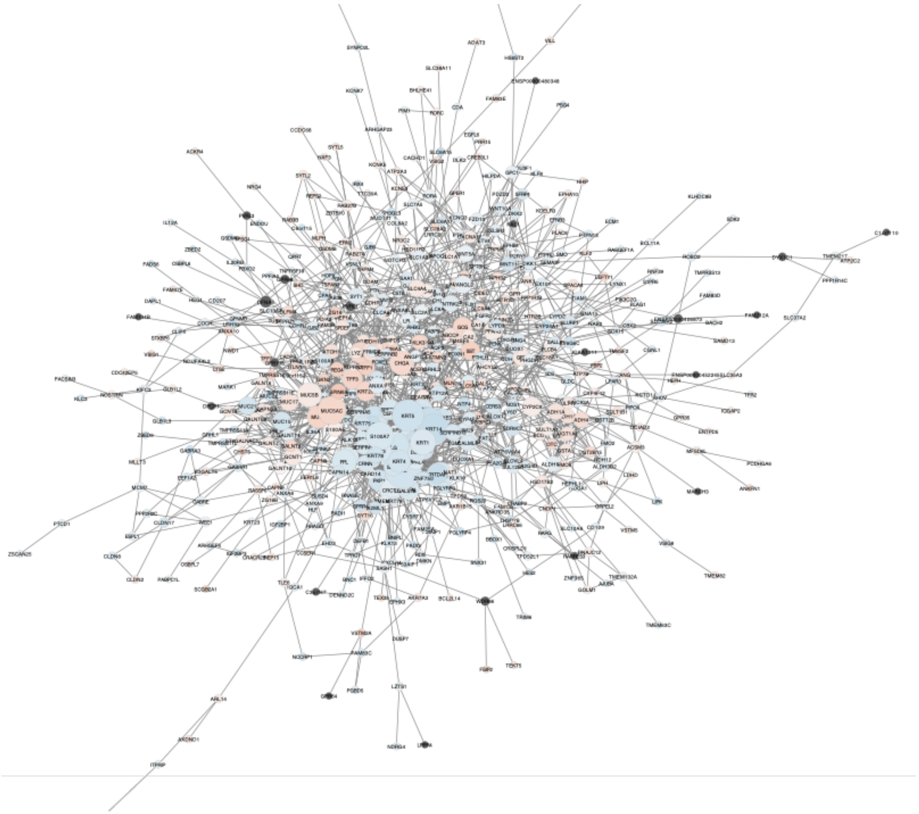

Figure S2: Comprehensive protein–protein interaction network analysis of differentially expressed genes (DEGs) in Barrett's esophagus (BE) vs. esophageal adenocarcinoma (EAC). Diagram illustrating the extensive network of protein–protein interactions among DEGs with |log2 fold-change (FC)| >2 in EAC vs. BE, highlighting the complex interplay occurring within the cellular environment during disease progression. Nodes represent individual proteins

encoded by the DEGs, and lines (edges) denote the known or predicted interactions between them. Node color intensity reflects the degree of upregulation (red) or downregulation (blue) and node sizes reflect the number of connections with other DEGs, providing a visual correlation between gene expression levels and network centrality. Central to this network is keratin 14 (KRT14), which was identified as a hub gene due to its numerous connections with both upregulated and downregulated genes. The prominence of KRT14 in this network underscores its potential regulatory role in the pathophysiology of EAC and identifies this protein as a key player in the cellular transition from BE to EAC.

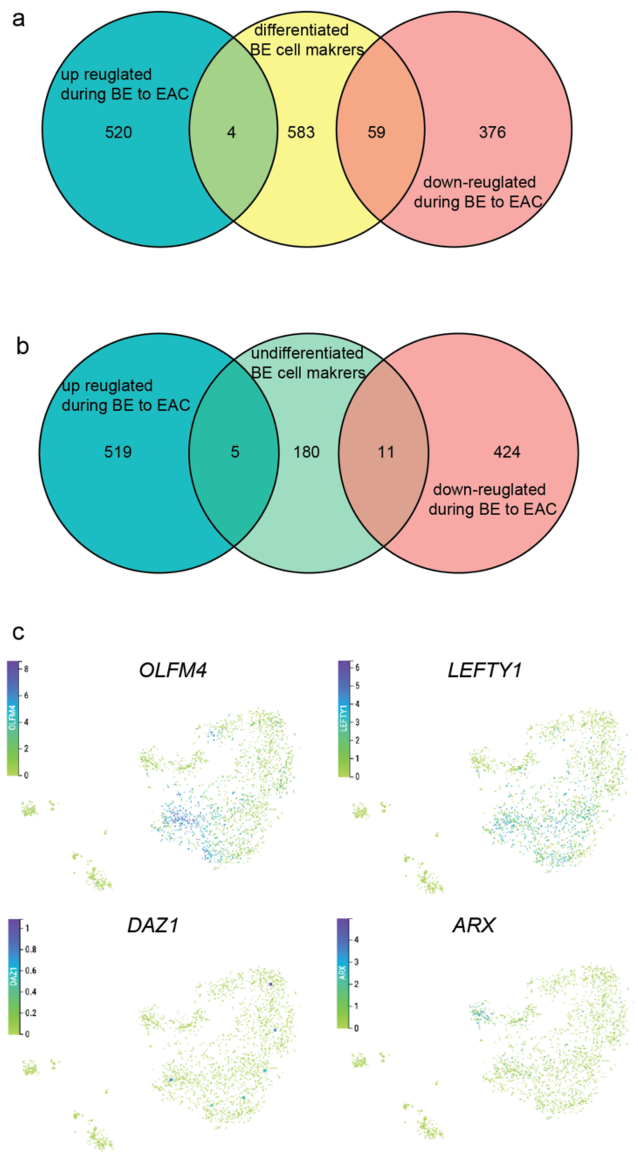

Figure S3. Expression changes in differentiated and undifferentiated BE cell marker genes during the progression from Barrett's esophagus (BE) to esophageal adenocarcinoma (EAC). (a,b) Venn diagram illustrating the differentiated (a) and undifferentiated (b) BE cell markers that are upregulated and downregulated during the BE-to-EAC transition. The left circles (blue) represent genes that are upregulated during the transition from BE to EAC, whereas the right circle (red) shows genes that are downregulated during the same transition. Overlapping genes with the differentiated and undifferentiated marker set are shown in the yellow and cyan

circles, respectively. (c) Scatter plots representing the expression levels of four genes (OLFM4, LEFTY1, DAZ1, ARX) across the cell populations. Each plot is color-coded based on expression levels from low (blue) to high (green), indicating the distribution and relative expression of these markers in the progression from BE to EAC. Expression of the different genes was obtained from single-cell RNA sequencing analysis of BE tissues [10].

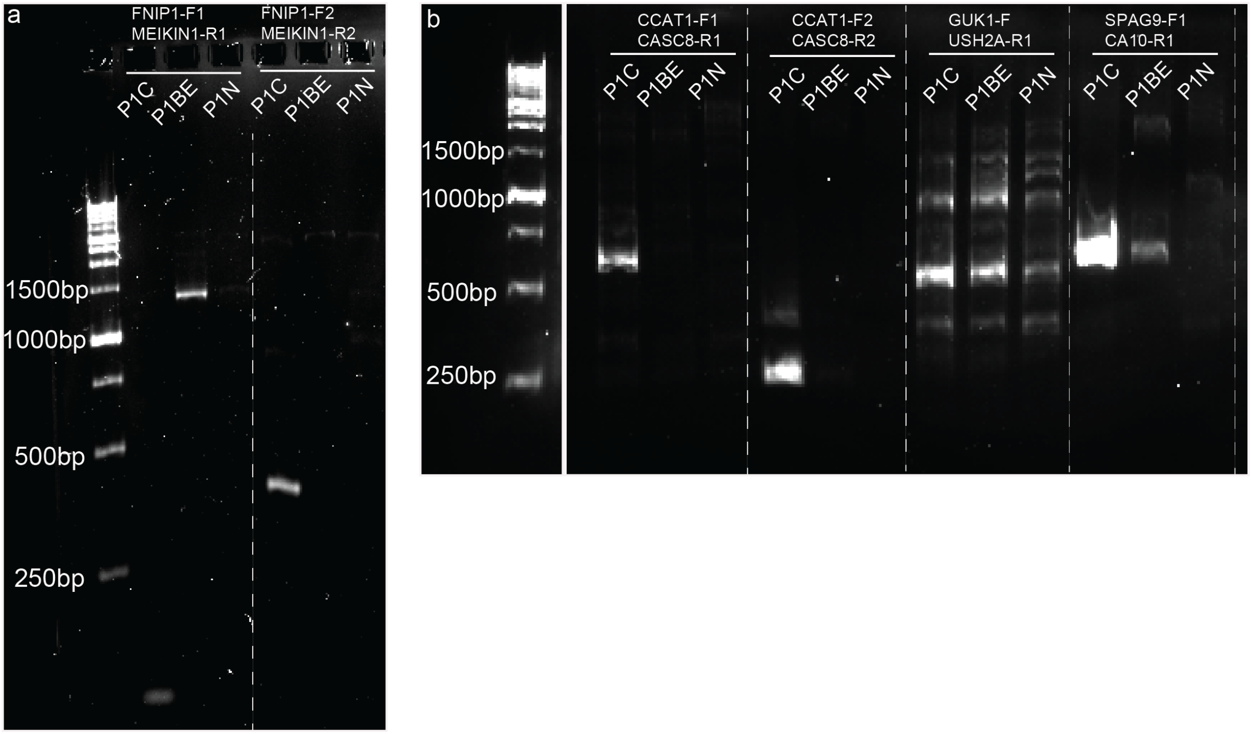

Figure S4: Reverse transcription (RT)-PCR confirming the existence of gene fusions predicted by the algorithm. There are paired EAC, BE, and normal tissues from the same individual (P1) to study the evolution of the suspected gene fusions. P1C: cancer, P1BE: Barrett, P1N: normal (a) RT-PCR with *FNIP1–MEIKIN* primer pairs validating the fusion between the

FNIP1 and *MEIKIN1* genes. The detailed junctions of this fusion were confirmed by whole-plasmid sequencing (see **Figure 4,** main text). The predicted fusions are only detected in esophageal adenocarcinoma (EAC) samples and are absent in Barrett's esophagus (BE) and normal samples. The band in P1-BE obtained using the FNIP1-F1 and MEIKIN1-R1 primers was found to be a non-specific binding product of the primers. (b) The *CCAT1–CASC8* fusion was detected by the indicated primer pairs and confirmed by whole-plasmid sequencing (see **Figure S4** below). The fusion is present only in cancer tissues and absent in BE and normal samples. We were unable to validate the *GUK1–USH2A* fusion. The *SPAG9–CA10* fusion was detected and validated by whole-plasmid sequencing (see **Figure S5** below). This fusion is present in both BE and EAC but shows a greater abundance in EAC tissues (100% EAC vs 10% BE). We did not detect a band in normal tissues.

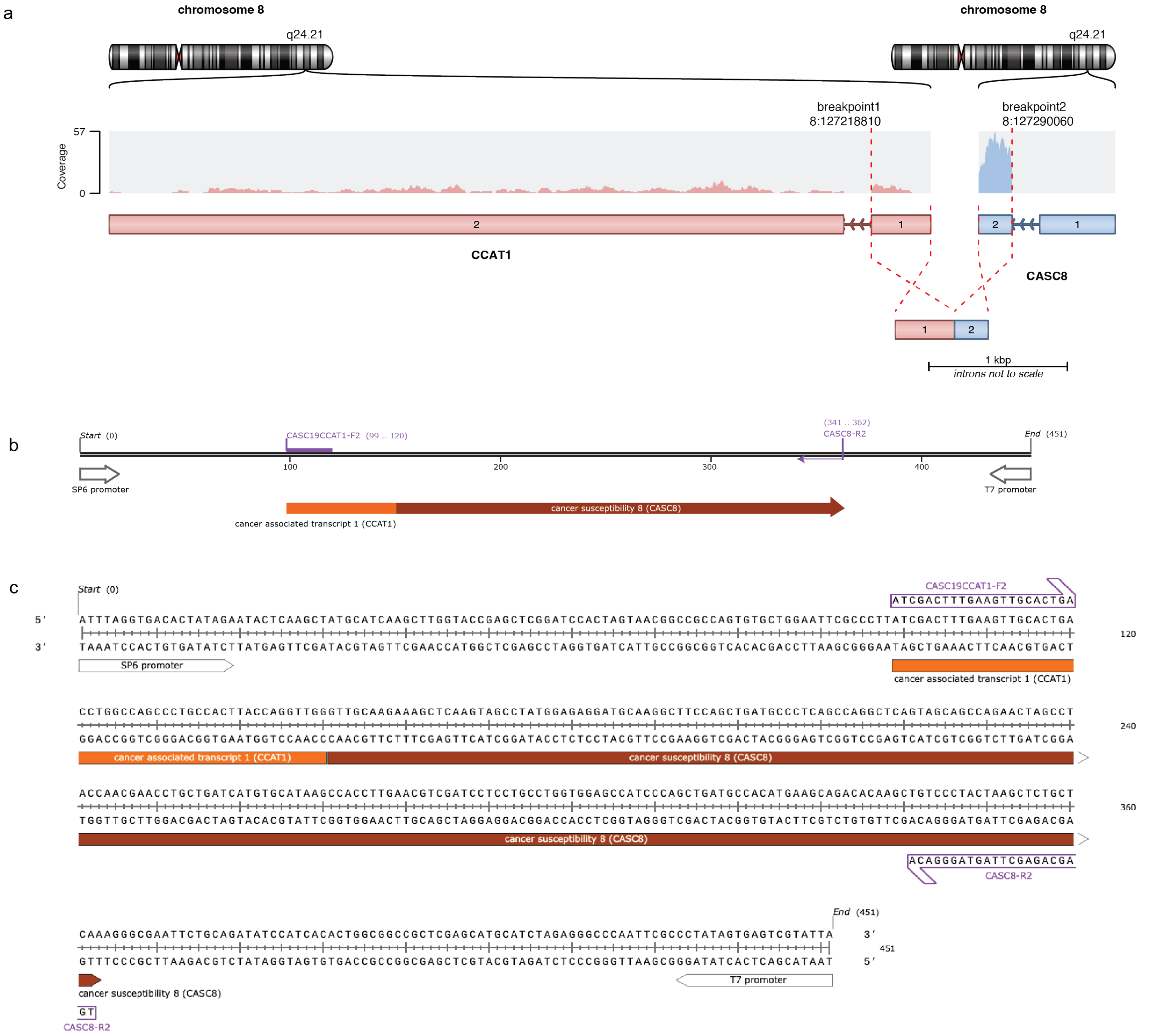

Figure S5. Analysis and validation of the predicted *CCAT1–CASC8* gene fusion predicted from RNA sequencing (RNA-seq) data. (a) Genomic mapping and expression profiling for the potential *CCAT1–CASC8* fusion. The top panel shows the chromosome 8 bands with the positions of the *CCAT1* and *CASC8* genes indicated. The middle panel illustrates the expression levels across exons of both genes, with a higher density of reads suggesting increased expression. The detailed exon structures for both genes are shown, with the direction of transcription indicated by arrows. Dotted lines between the genes indicate the predicted fusion points based on RNA-seq data. (b) The fusion gene structure as indicated by whole-plasmid

sequencing. The *CCAT1* gene (orange) was shown to be directly fused with the *CASC8* gene by the CCAT1-F2 and CASC8-R2 primer pairs. (c) Detailed sequences of the *CCAT1–CASC8* fusion illustrating the junction of the fused genes.

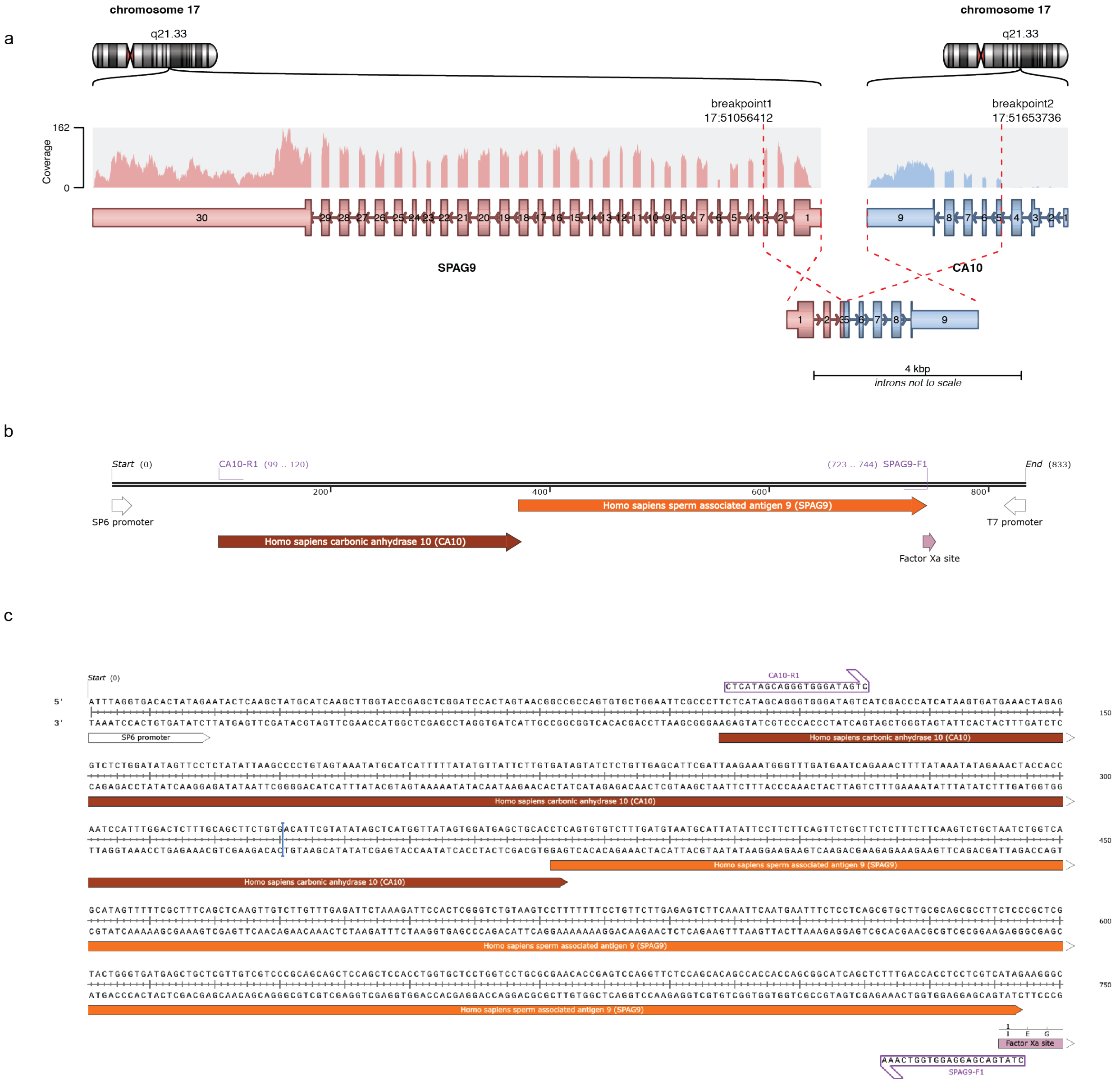

Figure S6. Analysis and validation of the predicted *SPAG9–CA10* gene fusion predicted from RNA sequencing (RNA-seq) data. (a) Genomic mapping and expression profiling for the potential *SPAG9–CA10* fusion. The top panel shows the chromosome 17 bands with the positions of the *SPAG9* and *CA10* genes indicated. The middle panel illustrates the expression levels across exons of both genes, with a higher density of reads suggesting increased expression. The detailed exon structures for both genes are shown, with the direction of transcription indicated by arrows. Dotted lines between the genes indicate the predicted fusion points based on RNA-seq data. (b) The fusion gene structure as indicated by whole-plasmid sequencing. The *SPAG9* gene (orange) was shown to be directly fused with the *CA10* gene by the SPAG9-F1 and CA10-R1 primer pairs. (c) Detailed sequences of the *SPAG9–CA10* fusion illustrating the junction of the fused genes.

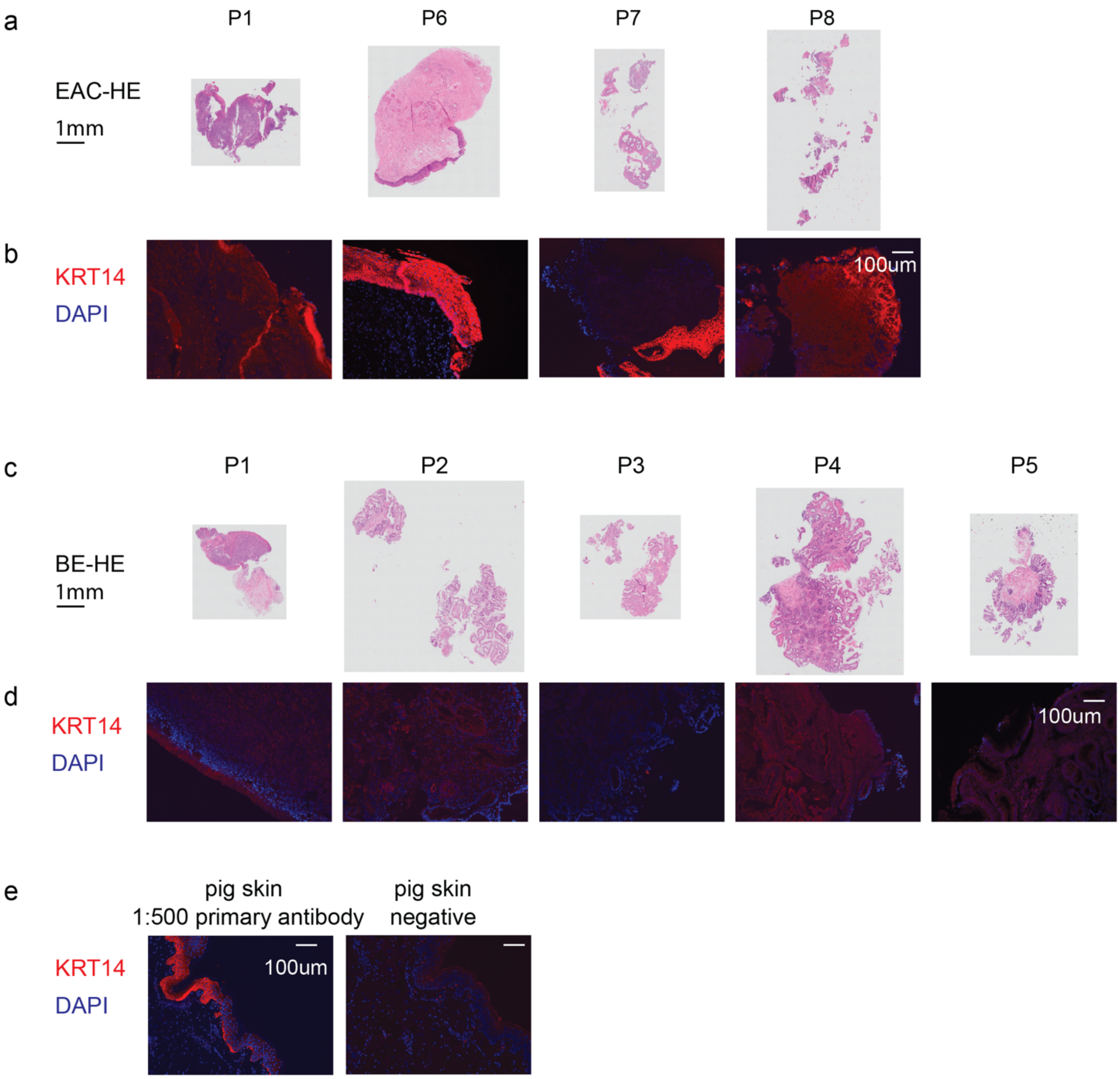

Figure S7. Elevated KRT14 protein levels in esophageal adenocarcinoma (EAC) compared to Barrett's esophagus (BE). Keratins are a family of intermediate filament proteins primarily found in epithelial cells [22] that play a pivotal role in maintaining cell structure and are involved in various cell signaling pathways [22]. Aberrant expression in keratin genes can disrupt cellular functions and contribute to cancer development [23]. One of the most striking findings of our

study is the significant alteration in the expression of keratin gene family members. Despite their known importance in the structural integrity of epithelial cells, the specific role of keratins in the development of EAC from BC remains largely unexplored. Our findings suggest a critical role for these genes, particularly KRT14 and KRT5, in the pathogenesis of EAC. (a) Hematoxylin and Eosin (H&E) staining of EAC tissue samples from patients P1, P6, P7, and P8 showing heterogeneity within the tumoral architecture. The images capture multiple views from each sample, providing a comprehensive assessment of the cellular morphology. (b) Immunofluorescence (IF) staining for keratin 14 (KRT14) in EAC samples from patients P1, P6, P7, and P8. KRT14 expression is markedly elevated in EAC samples. From same individuals as in Figure 4e but with different views. (c) H&E staining of BE tissue samples from patients P1-5 depicting the distinctive glandular structure of BE. (d) IF staining for KRT14 in BE samples from patients shown in P1-5, revealing low expression of KRT14 and highlighting the difference in protein expression profiles between EAC and BE tissues. From same individuals as in Figure 4f but with different views. (e) Control IF staining of pig skin tissue to validate KRT14 antibody specificity. This panel includes a positive control with 1:500 primary antibody and a negative control without primary antibody to delineate the KRT14-specific staining pattern compared to background staining.

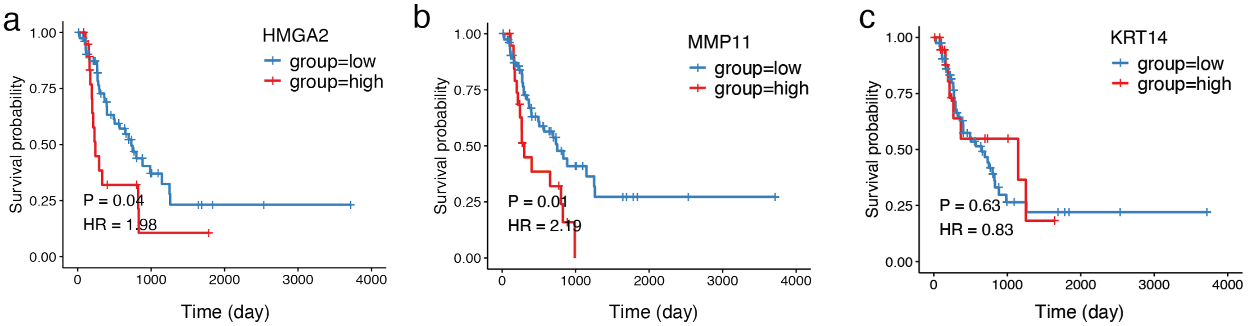

Figure S8. Additional biomarkers for worse EAC progression-free interval (PFI). Esophageal Adenocarcinoma patients from TCGA were analyzed for their PFI. High expression refers to the

top 20% expression of a gene of interest in the cohort. (**a**) Kaplan-Meier curve for patient with different HMGA2 expression levels. The blue line represents individuals with low HMGA2 expression, and the red line represents individuals with high HMGA2 expression. HMGA2 overexpression is known to lead to poor patient survival in EAC[24]. (**b-c**) Kaplan-Meier curves based on MMP11(**b**), and KRT14(**c**) expression. While high MMP11 predicts worse clinical outcome, high KRT14 does not. These suggest that there are more biomarkers to be investigated for disease progression and patient outcomes.

Supplementary Table 1: EAC/BE differentially expressed genes and statistics.

Supplementary Table 2: Gene Ontology (GO), Molecular Pathway (MP), Kyoto Encyclopedia of Genes and Genomes (KEGG) enrichment analysis complete layouts.

Supplementary Table 3: Gene fusion prediction for each specimen.

Supplementary Table 4: Primers used in this study.

Supplementary text 1: clinical relevance and biomarkers for EAC, limitations, and future directions.

The clinical relevance of this study stems from its potential to develop specific biomarkers, such as increased levels of keratin 14 (KRT14), for early detection of EAC in the BE stages, thereby improving prognosis and survival rates. Additionally, the identification of transcriptomic changes, such as the upregulation of keratin family genes, and gene fusions, like FNIP1–MEIKIN, during the progression from BE to EAC, could lead to targeted therapeutic strategies and personalized medicine. Although specific biomarkers of these gene fusions to predict EAC progression are not yet definitive, quantifying the gene fusion events overall are promising as a biomarker to predict BE-to-EAC transition. Tailoring treatment plans based on these specific molecular markers could significantly enhance treatment efficacy and patient outcomes in EAC.

Limitations: We acknowledge that our findings, though promising, are preliminary and require further validation. First, the sample size is small, which may impact the generalizability of the findings. Second, the transcriptional heterogeneity within the samples was not addressed due to the limitations of bulk RNA-seq approaches. Lastly, the mechanisms and consequences of the identified gene fusion events remain unknown.

Future studies investigating the functional implications of these identified changes are also needed. In particular, understanding the impact of alterations in keratin family genes and oncogene fusions on cell behavior, as well as their contribution to disease progression, could provide critical insights into potential therapeutic targets and strategies for EAC. A potential therapeutic role for keratin inhibitors in slowing down the BE-to-EAC transition presents another promising area for future research. In vivo testing these inhibitors in model organisms, as well as in cellular subpopulations enriched from patient samples, could provide crucial insights into their efficacy and mechanism of action.

References

1. Corlett R, Button C, Scheel S, Agrawal S, Rai V, Nandipati KC: **miRNA profiling of esophageal adenocarcinoma using transcriptome analysis.** *Cancer Biomark* 2024;**39**(3):245-264

2. Patel H, Ewels P, Peltzer A, Manning J, Botvinnik O, Sturm G, Garcia MU, Moreno D, Vemuri P: **nf-core/rnaseq: nf-core/rnaseq v3. 14.0-Hassium Honey Badger.** *Zenodo* https://doi.org/10.5281/zenodo 2024, **10471647**.

3. Dobin A, Davis CA, Schlesinger F, Drenkow J, Zaleski C, Jha S, Batut P, Chaisson M, Gingeras TR: **STAR: ultrafast universal RNA-seq aligner.** *Bioinformatics* 2013, **29**(1):15-21.

4. Patro R, Duggal G, Love MI, Irizarry RA, Kingsford C: **Salmon provides fast and bias-aware quantification of transcript expression.** *Nat Methods* 2017, **14**(4)**:**417.

5. Love MI, Huber W, Anders S: **Moderated estimation of fold change and dispersion for RNA-seq data with DESeq2.** *Genome Biol* 2014, **15**(12):550-8.

6. Shannon P, Markiel A, Ozier O, Baliga NS, Wang JT, Ramage D, Amin N, Schwikowski B, Ideker T: **Cytoscape: A Software Environment for Integrated Models of Biomolecular Interaction Networks.** *Genome Res* 2003, **13**(11)**:**2498.

7. Szklarczyk D, Kirsch R, Koutrouli M, Nastou K, Mehryary F, Hachilif R, Gable AL, Fang T, Doncheva NT, Pyysalo S, Bork P, Jensen LJ, von Mering C: **The STRING database in 2023: protein-protein association networks and functional enrichment analyses for any sequenced genome of interest.** *Nucleic Acids Res* 2023, **51**(D1):D638-D646.

8. Wang J, Duncan D, Shi Z, Zhang B: **WEB-based GEne SeT AnaLysis Toolkit (WebGestalt): update 2013.** *Nucleic Acids Research* 2013, **41**(W1)**:**W77.

9. Wu X, Ajani JA, Gu J, Chang DW, Tan W, Hildebrandt MAT, Huang M, Wang KK, Hawk E: **MicroRNA Expression Signatures during Malignant Progression from Barrett's Esophagus to Esophageal Adenocarcinoma.** *Cancer Prevention Research* 2014, **6**(3)**:**196.

10. Nowicki-Osuch K, Zhuang L, Jammula S, Bleaney CW, Mahbubani KT, Devonshire G, Katz-Summercorn A, Eling N, Wilbrey-Clark A, Madissoon E, Gamble J, Di Pietro M, O'Donovan M, Meyer KB, Saeb-Parsy K, Sharrocks AD, Teichmann SA, Marioni JC, Fitzgerald RC: **Molecular phenotyping reveals the identity of Barrett's esophagus and its malignant transition.** *Science* 2021, **373**(6556):760-767.

11. Wang Z, Cheng Y, Abraham JM, Yan R, Liu X, Chen W, Ibrahim S, Schroth GP, Ke X, He Y, Meltzer SJ: **RNA sequencing of esophageal adenocarcinomas identifies novel fusion transcripts, including NPC1-MELK, arising from a complex chromosomal rearrangement.** *Cancer* 2017, **123**(20):3916-3924.

12. Kim J, Ishiguro K, Nambu A, Akiyoshi B, Yokobayashi S, Kagami A, Ishiguro T, Pendas AM, Takeda N, Sakakibara Y, Kitajima TS, Tanno Y, Sakuno T, Watanabe Y: **Meikin is a conserved regulator of meiosis-I-specific kinetochore function.** *Nature* 2015, **517**(7535):466-471.

13. Haas BJ, Dobin A, Li B, Stransky N, Pochet N, Regev A: **Accuracy assessment of fusion transcript detection via read-mapping and de novo fusion transcript assembly-based methods.** *Genome Biol* 2019, **20**(1):213-9.

14. Uhrig S, Ellermann J, Walther T, Burkhardt P, Frohlich M, Hutter B, Toprak UH, Neumann O, Stenzinger A, Scholl C, Frohling S, Brors B: **Accurate and efficient detection of gene fusions from RNA sequencing data.** *Genome Res* 2021, **31**(3):448-460.

15. Daniel Nicorici, Mihaela Şatalan, Henrik Edgren, Sara Kangaspeska, Astrid Murumägi, Olli Kallioniemi, Sami Virtanen, Olavi Kilkku: **FusionCatcher – a tool for finding somatic fusion genes in paired-end RNA-sequencing data.** *bioRxiv* 2014, :011650.

16. Bray NL, Pimentel H, Melsted P, Pachter L: **Near-optimal probabilistic RNA-seq quantification.** *Nat Biotechnol* 2016, **34**(5):525-527.

17. Páll Melsted, Shannon Hateley, Isaac Charles Joseph, Harold Pimentel, Nicolas Bray, Lior Pachter: **Fusion detection and quantification by pseudoalignment.** *bioRxiv* 2017, :166322.

18. Haas BJ, Dobin A, Ghandi M, Van Arsdale A, Tickle T, Robinson JT, Gillani R, Kasif S, Regev A: **Targeted in silico characterization of fusion transcripts in tumor and normal tissues via FusionInspector.** *Cell Reports Methods* 2023, **3**(5)**:**100467

19. Holmes TR, Al Matouq J, Holmes M, Sioda N, Rudd JC, Bloom C, Nicola L, Palermo NY, Madson JG, Lovas S, Hansen LA: **Targeting 14-3-3epsilon activates apoptotic signaling to prevent cutaneous squamous cell carcinoma.** *Carcinogenesis* 2021, **42**(2):232-242.

20. Smathers RL, Chiang DJ, McMullen MR, Feldstein AE, Roychowdhury S, Nagy LE: **Soluble IgM links apoptosis to complement activation in early alcoholic liver disease in mice.** *Mol Immunol* 2016, **72**:9-18.

21. Goldman MJ, Craft n, Hastie M, Repecka K, McDade F, Kamath A, Banerjee A, Luo Y, Rogers D, Brooks AN, Zhu J, Haussler D: **Visualizing and interpreting cancer genomics data via the Xena platform.** *Nat Biotechnol* 2020, **38**(6):675-678.

22. Lam VK, Sharma P, Nguyen T, Nehmetallah G, Raub CB, Chung BM: **Morphology, Motility, and Cytoskeletal Architecture of Breast Cancer Cells Depend on Keratin 19 and Substrate.** *Cytometry Pt A* 2021, **97**(11)**:**1145.

23. Karantza V: **Keratins in health and cancer: more than mere epithelial cell markers.** *Oncogene* 2011, **30**(2):127-138.

24. Mito JK, Agoston AT, Dal Cin P, Srivastava A: **Prevalence and significance of HMGA2 expression in oesophageal adenocarcinoma.** *Histopathology* 2017, **71**(6):909-917.
